# Diagnostic and prognostic value of circulating tumor DNA in gastric cancer: a meta-analysis

**DOI:** 10.18632/oncotarget.14064

**Published:** 2016-12-21

**Authors:** Yunhe Gao, Kecheng Zhang, Hongqing Xi, Aizhen Cai, Xiaosong Wu, Jianxin Cui, Jiyang Li, Zhi Qiao, Bo Wei, Lin Chen

**Affiliations:** ^1^ Chinese PLA General Hospital, Department of General Surgery, Beijing, 100853, People's Republic of China

**Keywords:** ctDNA, gastric cancer, diagnosis, prognosis, meta-analysis

## Abstract

**Background:**

Circulating tumor DNA (ctDNA) has offered a minimally invasive approach for detection and measurement of gastric cancer (GC). However, its diagnostic and prognostic value in gastric cancer still remains unclear.

**Results:**

A total of 16 studies comprising 1193 GC patients met our inclusion criteria. The pooled sensitivity and specificity were 0.62 (95% confidence intervals (CI) 0.59−0.65) and 0.95 (95% CI 0.93–0.96), respectively. The AUSROC (area under SROC) curve was 0.94 (95% CI 0.89–0.98). The results showed that the presence of certain ctDNA markers was associated with larger tumor size (OR: 0.26, 95% CI 0.11–0.61, *p* = 0.002), TNM stage (I + II/III + IV, OR: 0.11, 95% CI 0.07−0.17, *p* = 0.000), as well as *H. pylori* infection. (*H.p* negative/*H.p* positive, OR: 0.57, 95% CI 0.36–0.91, *p* = 0.018). Moreover, there was also a significant association between the presence of ctDNA and worse overall survival (HR 1.77, 95% CI 1.38−2.28, *p* < 0.001), as well as disease-free survival (HR 4.36, 95% CI 3.08−6.16, *p* < 0.001).

**Materials and Methods:**

Pubmed, Embase, Cochrane Library and Web of Science databases were searched for relating literature published up until November 30, 2016. Diagnostic accuracy variables were pooled by the Meta-Disc software. Engauge Digitizer and Stata software were applied for prognostic data extraction and analysis.

**Conclusions:**

Our meta-analysis indicates the detection of certain ctDNA targets is significantly associated with poor prognosis of GC patients, with high specificity and relatively moderate sensitivity.

## INTRODUCTION

Gastric cancer (GC) remains the fourth most common cancer and the second leading cause of cancer-related death in the world [1]. Although recent achievements in cancer diagnosis and therapy strategies have improved the clinical outcomes, a total of 950,000 new GC cases and 720,000 deaths related to GC were estimated to occur in 2014 worldwide [2]. Many patients are diagnosed with GC in its advanced stage due to the lack of early diagnostic techniques. Failure to identify patients with high-risk of metastasis and recurrence has also resulted in an unsatisfactory prognosis of GC patients.

Tumorigenesis and cancer progression involves a series of biological processes. During these complex events, cell-free DNA (cfDNA) might be released into the bloodstream by cells undergoing apoptosis or necrosis, as well as by exosomes [3]. The cfDNA derived from tumors, also known as circulating tumor DNA (ctDNA), contains different fragments of tumor gene, which reflect specific genetic alterations of cancer, such as methylation or mutation. These molecular alterations are analyzed by various strategies, including polymerase chain reaction followed by sequencing analyses or by methylation-specific PCR and digital PCR [4, 5]. Other researchers have also demonstrated the value of specific ctDNA in diagnosis or as a prognosis indicator for cancer. In non-small cell lung cancer (NSCLC), colon cancer, breast cancer, and other malignancies, the presence of certain ctDNA markers was found to be an indicator and predictor of tumor progression or drug resistance [6, 7]. Although many recent studies have focused on the relationship between cfDNA/ctDNA and GC, the results are still unclear. Therefore, this study initiated a comprehensive analysis to clarify the precise value of ctDNA in GC patient diagnosis and prognosis.

## RESULTS

### Study selection process

**Figure 1:**
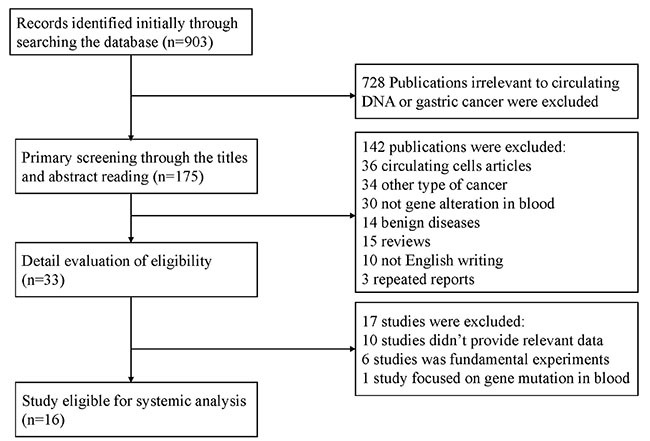
Flow chart of selection process to enroll eligible studies

**Figure 2:**
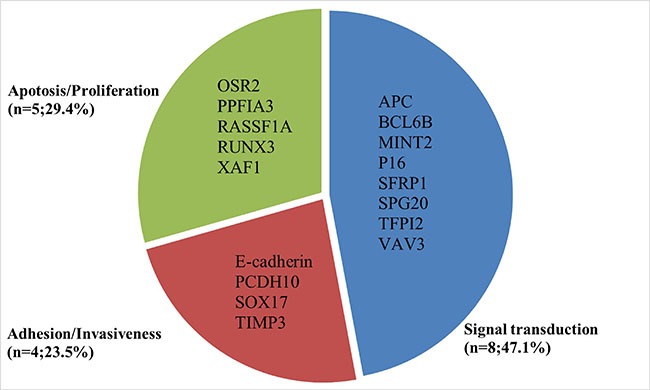
Summarized genetic alterations arranged by main gene function

**Figure 3:**
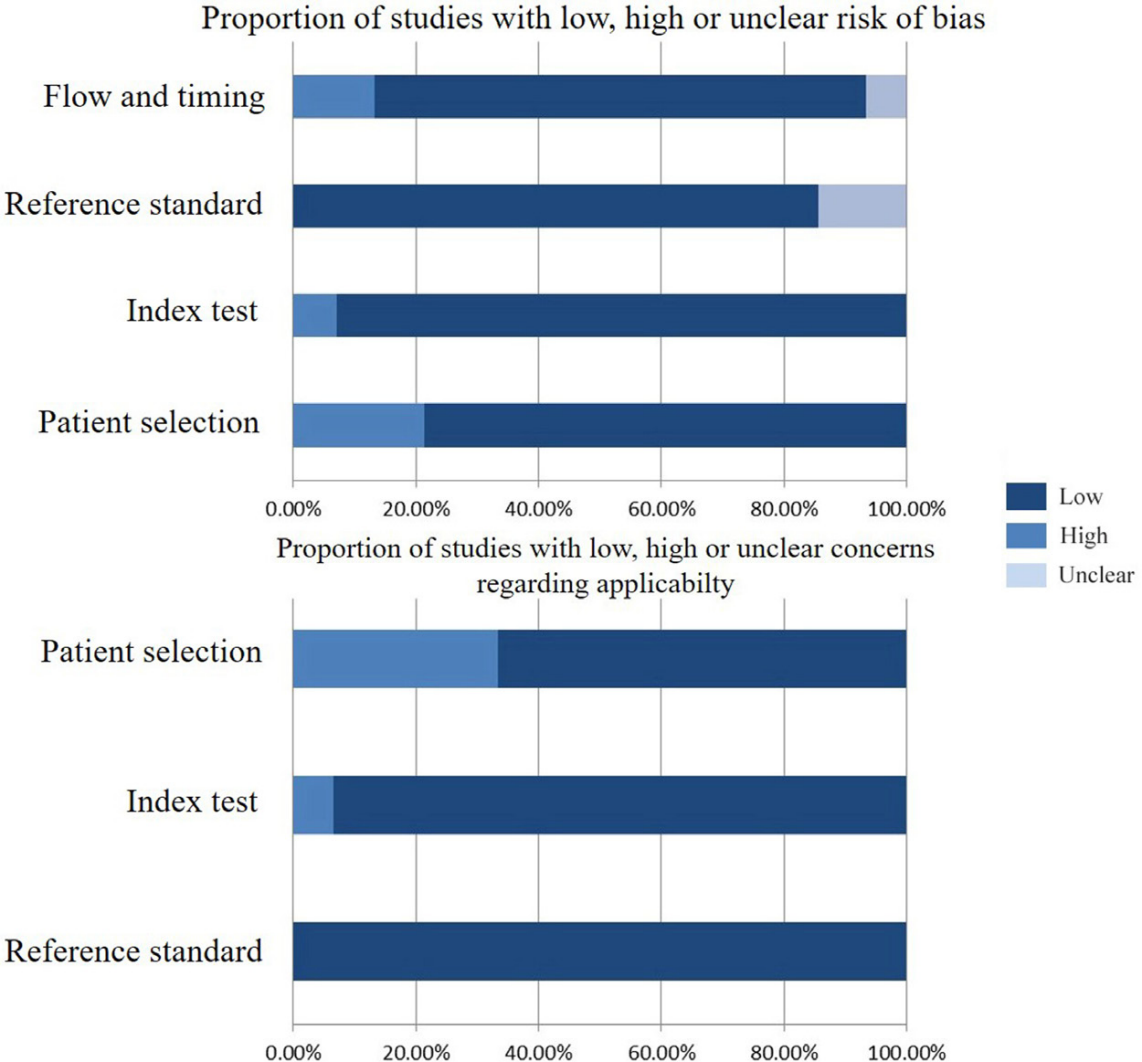
Diagnosis quality assessments of included studies using the QUADAS-2 tool criteria

As shown in Figure 1, 16 studies were eligible for system review after carefully screening and re-checking by the entire research group. The details and main characteristics of included studies are summarized in Table 1. The 16 eligible studies contained a total of 1193 patients with a median sample size of 69 (range: 40–202, mean: 73) and were published between 2005 and 2016. Among them, 13 studies enrolled patients from East Asian countries/areas (one from Hong Kong, one from Thailand, two from Japan and the remaining 10 from People's Republic of China). Two studies were performed in Greece and one was in Iran. The two studies from the same medical center in Greece (University General Hospital of Alexandropolis) involved different gene targets (SOX17, APC and RASSF1A); therefore, we considered them as independent studies. The genes in the analysis and their main function were classified according to Simone M's research [24] (Figure 2) and other detail information is showed in Supplementary Table S1. The results of diagnosis quality assessment are showed in Figure 3 and the detail information was displayed in Supplementary Table S2. The results of quality assessment according to NOS scale were presented in Supplementary Table S3 and all studies have achieved a score over 5 stars.

**Table 1:** Major characteristics of enrolled studies

No.	Study	Number	Sex (M/F)	region	Detection method	Target gene	HR	Follow up	AT	SS	ST	BV (ml)
1	WK Leung^8^	60	/	Hongkong	MSP	APC/E-cadherin	OS:3.38(1.42-8.05) (F)	8 (0–40)	Methylation	Serum	BS	0.8
2	Mohammad R.A.^9^	52	38/14	Iran	MSP	P16	/	/	Hypermethylation	Serum	BS	/
3	Wang YC^10^	47	29/18	China	MSP	RASSF1A	/	/	Hypermethylation	serum	BS	5
4	Chouhei.S^11^	65	37/28	Japan	qMSP	RUNX3	/	/	Methylation	Serum	BS	/
5	Kenji H.^12^	73	57/16	Japan	qMSP	TFPI2	/	/	Methylation	Serum	TOS	/
6	Ioanna B.^13^	73	51/22	Greece	MSP	SOX17	OS:1.60 (1.0–2.55) (F)	56 (20–111)	Methylation	Serum	BS	/
7	Yang QF^14^	40	33/7	China	BGS	BCL6B	OS:1.86 (0.68–5.10) (K)	/	Hypermethylation	Plasma	BS	1
8	Zhi QL^15^	202	120/82	China	MSP	XAF1	DFS:5.71 (3.474–9.383) (K)	/	Methylation	Serum	BS	/
9	Han J^16^	92	53/39	China	qMSP	MINT2	DFS:3.362 (1.779–5.981) (D)	/	Methylation	Serum	BS	/
10	Wu YC	92	53/39	China	qMSP	P16	DFS: 2.31 (1.00–5.37) (K)	/	Methylation	Serum	BS	/
11	Yu JL^17^	92	54/38	China	MSP	TIMP-3	DFS:97.376 (8.388–1130.378) (D)	/	Methylation	Serum	BS	/
12	Zhang H^18^	41	30/11	China	MSP	Spastic paraplegia-20	/	/	Hypermethylation		BS	2
13	Chang L^19^	42	30/12	China	MSP	SFRP1	/	/	Methylation	Serum	BS	5
14	Ioanna B. (APC)^20^	73	51/22	Greece	MSP	APC	OS: 2.94(1.33-6.53) (F)	56 (12–111)	Methylation	Serum	BS	/
	Ioanna B.(RASSF1A)^20^	73	51/22	Greece	MSP	RASSF1A	OS: 0.87(0.46-1.66) (F)	56 (12–111)	Methylation	Serum	BS	/
15	Charinya P (PCDH10)^21^	101	44/57	Tailand	MSP	PCDH10	OS:3.47(1.69-7.11) (F)	/	Methylation	plasma	BS	/
	Charinya P (RASSF1A) ^21^	101	44/57	Thailand	MSP	RASSF1A	OS:1.66(0.98-2.83) (F)	/	Methylation	plasma	BS	/
16	Li WH^22^	48 (25)	39/9	China	MSP	OSR2:VAV3:PPFIA3	/	/	Methylation	Serum	BS	0.4

K: extracted and calculated from the Kaplan-Meier curves in the studies; F: calculated by the formula provided by Parmar et al^37^. D: directly extracted by the authors in the studies; HR: hazard ratio; AT: alteration type; SS: sample source; ST: sample time; BV: blood volume; MSP: methylation-specific PCR; qMSP: quantitative methylation-specific PCR; BS: before surgery; TOS: time of surgery; OS: overall survival; DFS: disease-free survival.

### Detection of ctDNA

Several molecular detection methods, such as methylation-specific PCR (MSP) or quantitative MSP (qMSP), were applied in enrolled studies. One study [15] used bisulfite genomic sequence and another [23] used Taqman PCR to measure the status of specific circulating DNA in GC patients. The blood volume used for DNA detection varied between 400 ul–5 ml with a median volume of 1.5ml. All the studies collected blood samples before surgery except Henji's study, which withdrew patients’ blood during surgery. Fourteen out of all studies extracted DNA from patient serum and the other two from plasma. All the studies focused on circulating gene methylation/hypermethylation.

### Meta-analysis results of diagnostic value

**Figure 4:**
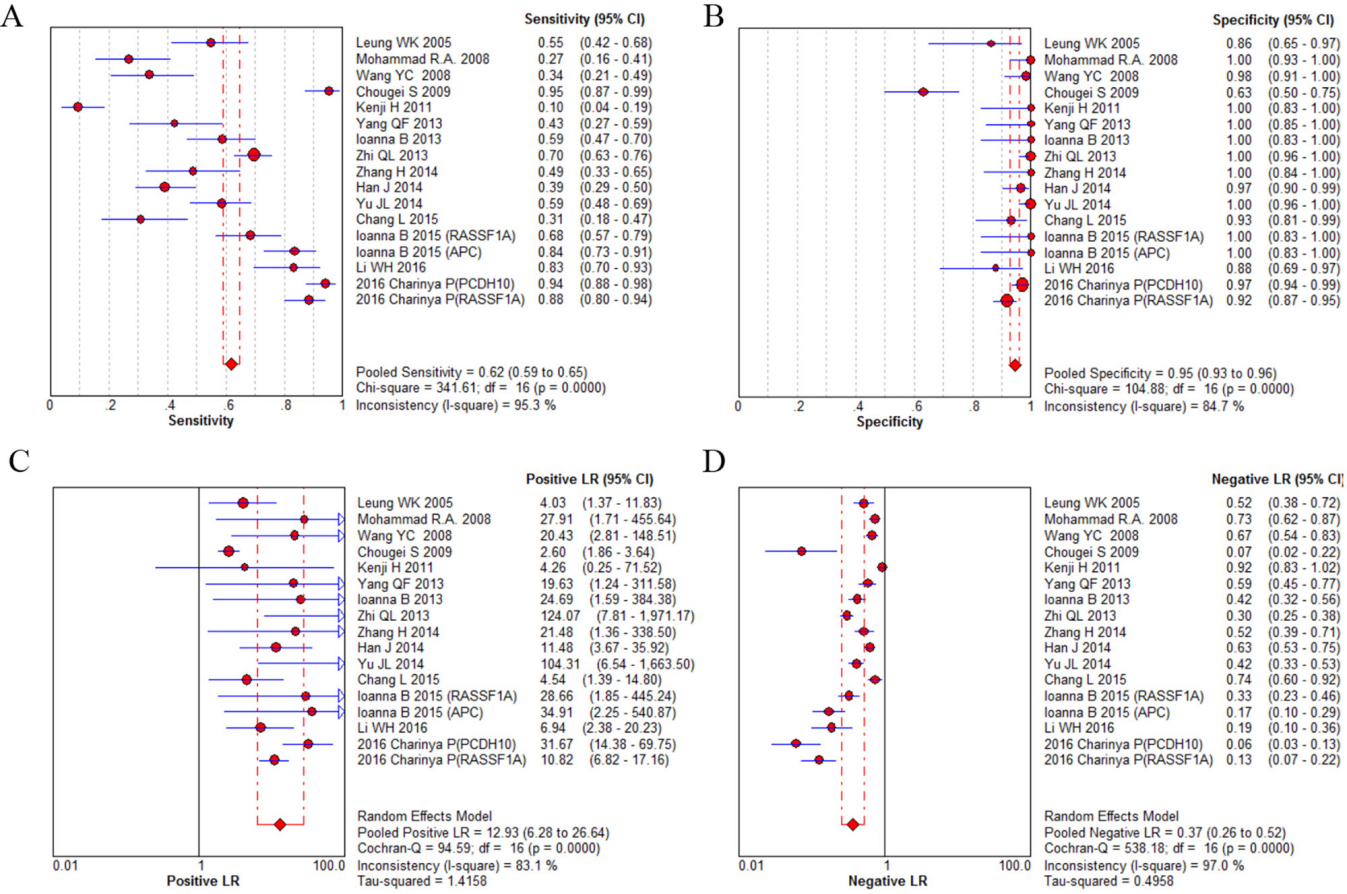
Diagnostic accuracy forest plots (**A**) Forest plots of overall sensitivity. (**B**) Forest plots of overall specificity. (**C**) Forest plots of positive likelihood ratio. (**D**) Forest plots of negative likelihood ratio.

Fifteen studies were pooled for the meta-analysis of diagnostic accuracy. As presented in Figure 4, the overall sensitivity and specificity was 0.62 (95% CI 0.59–0.65) and 0.95 (95% CI 0.93–0.96), respectively. The pooled PLR and NLR were 12.93 (95% CI 6.28–26.64) and 0.37 (95% CI 0.26–0.52), respectively. The area under the SROC was 0.94 (95% CI 0.89–0.98) and the DOR was 28.7 (95%CI 15.09–54.67). Significant heterogeneity was observed in the diagnostic analysis of 15 studies (sensitivity: *I^2^* = 95.3%, *p* = 0.000; specificity: *I^2^* = 84.7%, *p* = 0.000). Analysis of diagnostic threshold showed no significant threshold effect existed with the Spearman correlation coefficient of 0.39 and *p* value of 0.122. Therefore, subgroup analysis (Table 2A) was conducted according to different parameters: sample size (≥ 65 versus < 65), sample source (plasma versus serum), race (Caucasian versus Asian), and gene targets (single versus combined). However, only a lower heterogeneity was detected in the subgroup of Caucasian race (specificity: *I^2^* = 0%, *p* = 1.0) and single gene target (specificity: *I^2^* = 0%, *p* = 0.867). In the subgroup analysis for DOR, similar trend was also found in the qPCR group and Caucasian race (qPCR: *I^2^* = 0%, *p* = 0.413; Caucasian race: *I^2^* = 0%, *p* = 0.870). These results suggested that ctDNA detection method, race and gene target's combination type might be part of the heterogeneity source. The other measures of diagnosis value for subgroup analysis are summarized in A. Meta-regression analysis based on those four factors were also applied to explore the heterogeneity source. However, none of those factors would significantly alter the heterogeneity of universal diagnostic value. (Table 2B) Taken together, we considered race, detection method and gene targets combination as part of the heterogeneity source. And more well-design experiments with consistent methodology in different races are needed to clarify ctDNA's diagnostic role in GC patients.

**Figure 5:**
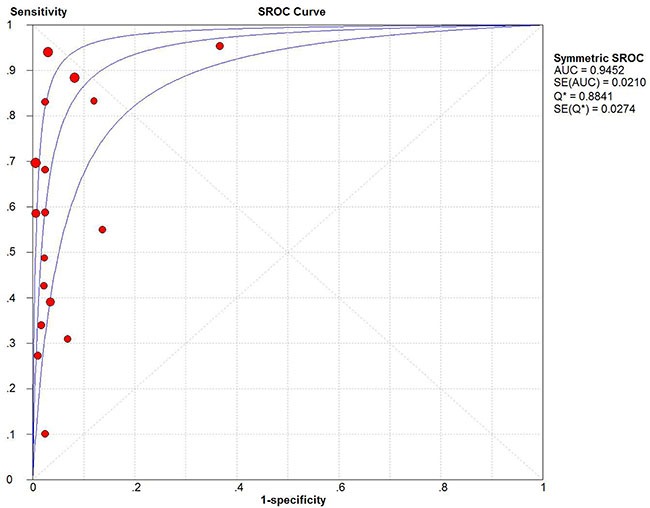
Summary receiver operating characteristic plot for the included studies with the associated 95% confidence region

**Table 2A:** Subgroup analysis of diagnosis measures

Subgroup	Sensitivity			Specificity			Diagnostic ratios		
	Value	*I*^2^ (%)	*P*	Value	*I*^2^ (%)	*P*	Value	*I*^2^ (%)	*P*
Method									
MSP	0.66 (0.63–0.69)	93.3	0.000	0.96 (0.95–0.97)	70.6	0.0001	58.16 (23.44–144.29)	69.3	0.0001
qPCR	0.46 (0.39–0.52)	98.4	0.000	0.85 (0.79–0.90)	94.5	0.000	21.82 (9.38–50.76)	0.0	0.413
Race									
Mongolian	0.62 (0.59–0.65)	96.0	0.000	0.94 (0.92–0.95)	86.9	0.000	39.82 (17.02–93.15)	72.5	0.000
Caucasian	0.62 (0.56–0.68)	93.2	0.000	1.00 (0.97–1.00)	0.0	1.000	79.01 (18.97–328.98)	0.0	0.870
Size									
< 65	0.46 (0.41–0.52)	86.9	0.000	0.96 (0.93–0.98)	61.4	0.017	15.43 (7.89–30.19)	0.0	0.431
≥ 65	0.67 (0.64–0.70)	96.4	0.000	0.94 (0.92–0.96)	89.8	0.000	80.92 (32.86–199.28)	61.3	0.006
Gene target									
Single	0.68 (0.58–0.76)	90.2	0.001	0.87 (0.74–0.95)	0.0	0.867	16.45 (3.57–75.79)	59.6	0.116
Combined	0.61 (0.59–0.64)	95.8	0.000	0.95 (0.93–0.96)	86.1	0.000	53.31 (24.26–117.13)	61.8	0.0008

*I*^2^ = inconsistency index; MSP = methylation-specific PCR; qMSP = quantitative methylation-specific PCR.

**Table 2B:** Meta-regression results of diagnostic value

Parameter	Sensitivity			Specificity		
	Coef	Z	*P*	Coef	Z	*P*
Method	–0.03	–0.70	0.49	3.65	–0.21	0.83
Race	0.44	–0.04	0.97	30.10	0.00	1.00
Size	1.21	0.88	0.38	4.42	0.31	0.76
Gene target	2.07	2.37	0.02	2.74	–1.09	0.27

Meta-analysis results of prognostic significance

**Figure 6:**
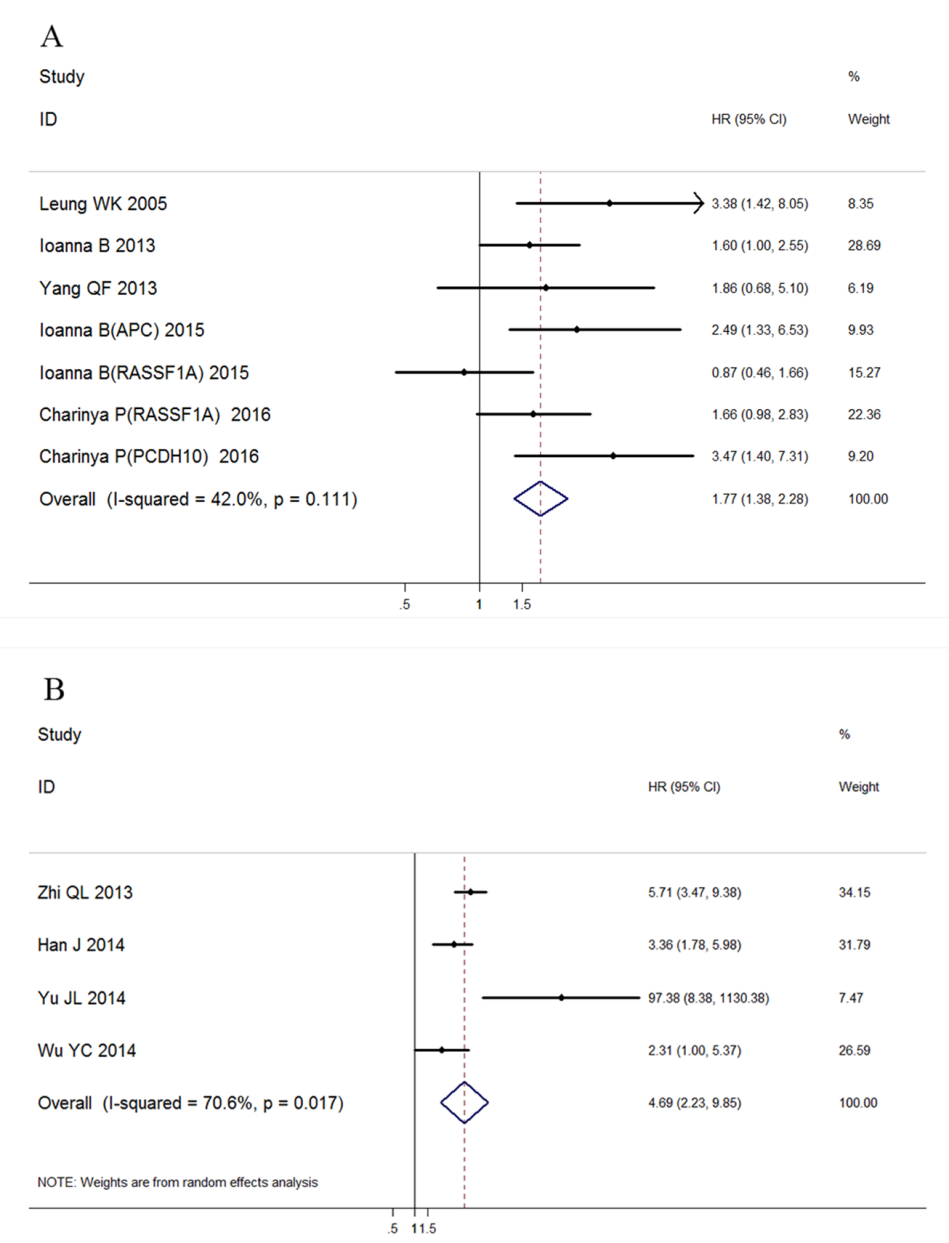
Forest plot of the HRs for survival in ctDNA detection of GC patients (**A**) Association with overall survival; (**B**) Association with disease free survival.

A total of 10 studies were pooled for meta-analysis of survival. Among them, 6 studies were available for calculating overall survival and 4 for disease-free survival. The results showed that high level of ctDNA in GC was associated with worse overall survival (HR: 1.77, 95%CI 1.38–2.28, *p* < 0.001). A moderate but insignificant heterogeneity was observed (*I^2^* = 42.0%, *p* = 0.111), so a fixed-effect model was applied during calculation (Figure 6A).

**Figure 7:**
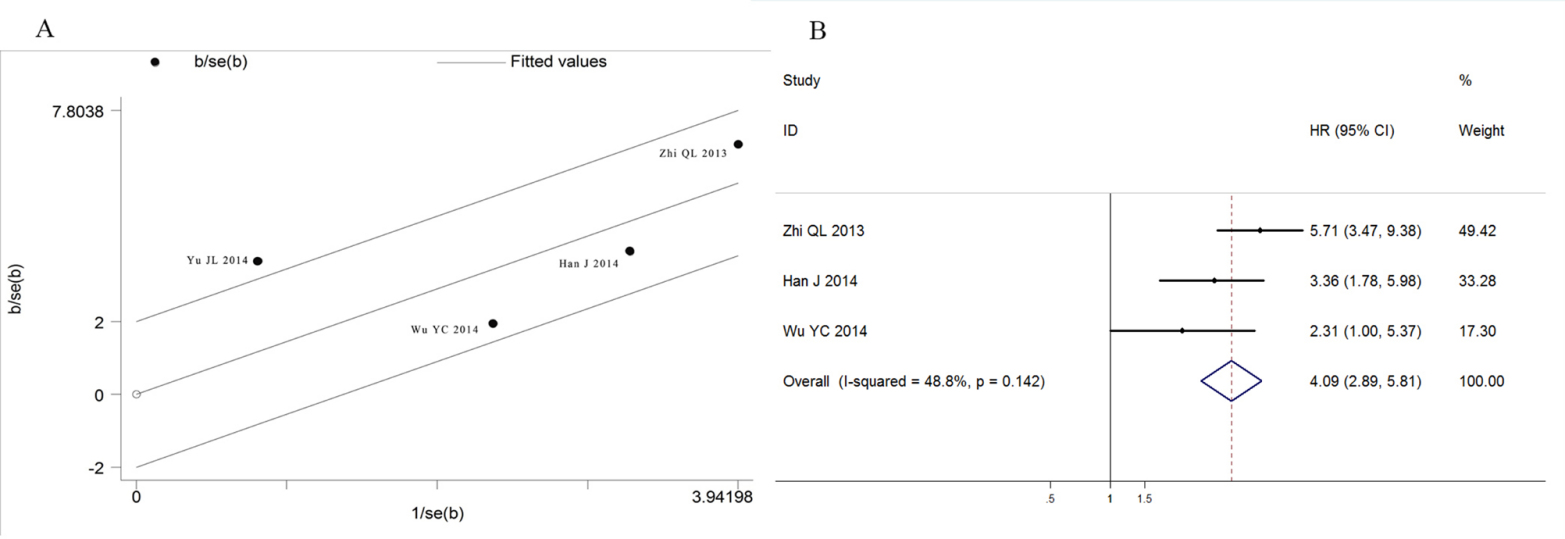
Heterogeneity exploration in DFS analysis (**A**) Galbraith blot of association between ctDNA and disease free survival; (**B**) Forest plot of HRs for disease free survival after omission of Yu JL's study.

Similarly, a significant association was found between ctDNA presence and GC patients' disease-free survival (HR: 4.69, 95%CI 2.23–9.85, *p* < 0.001) with a detectable heterogeneity (Figure 6B, I2 = 70.6%, *p* = 0.017). Galbraith plot was performed to explore the source of heterogeneity, and the results showed that Yu JL's study might cause the study heterogeneity (Figure 7A). After removing Yu JL's study, heterogeneity for DFS analysis decreased to an insignificant level (*I^2^* = 48.8%, *p* = 0.142), while the association between ctDNA and DFS remain significant (Figure 7B, HR:4.09, 95% CI 2.89–5.81, *p* < 0.001).

### Association of ctDNA detection with clinicopathological characteristics of GC patients

The association between ctDNA detection and major clinicopathological features was assessed using 12 studies. As shown in Table 3, ctDNA presence was significantly associated with TNM stage (I+II/III+IV, OR: 0.11, 95% CI 0.07–0.17, *p* = 0.000). In detail, ctDNA presence had a significant association with tumor depth (I + II/III + IV, OR: 0.18, 95% CI 0.07–0.45, *p* = 0.001), more lymph node metastasis (N0/N1-3, OR: 0.19, 95% CI 0.06–0.64, *p* = 0.008), as well as distant metastasis (M0/M1, OR: 0.32, 95% CI 0.20–0.53, *p* < 0.001). A significant association was also deteced between *H. pylori* infection and ctDNA presence. (*H.p* negative/*H.p* positive, OR: 0.57, 95% CI 0.36–0.91, *p* = 0.018). GC patients with larger tumor load were more likely to have detectable ctDNA (tumor size < 5 cm/> 5 cm, OR: 0.26, 95 CI 0.11–0.61, *p* = 0.002). Meanwhile, no statistical association was observed between ctDNA and sex (male/female, OR: 1.11, 95% CI 0.84–1.46, *p* = 0.476), Lauren classification (intestinal/diffuse, OR: 0.89, 95% CI 0.57–1.4, *p* = 0.628)

**Table 3:** Meta-analysis of the association between ctDNA presence and clinicopathological features of GC patients

Stratification	No. of studies	No. of patients	Pooled OR	95% CI of pooled OR	*P* value	Heterogeneity	
						I^2^ (%)	*P*-value
SEX (M/F)	12	876	0.97	0.72-1.31	0.849	0	0.763
pT (I + II/III + IV)	8	545	0.18	0.07-0.45	0.001	79.6	0.000
Lymph node metastasis (N0/N1-3)	7	744	0.19	0.06-0.64	0.008	90.2	0.000
Distant metastasis (M0/M1)	7	606	0.32	0.20-0.53	0..000	41.5	0.072
TNM stage(I + II/III + IV) a	6	561	0.11	0.07-0.17	0.000	90.4	0.000
Tumor size (< 5 cm/< 5 cm)	4	665	0.26	0.11-0.61	0.002	79.9	0.002
Lauren's classification (Intestinal/Diffuse)	2	317	0.89	0.57-1.4	0.628	0.000	0.808
H. pylori infection (Negative/positive)	3	386	0.57	0.36-0.91	0.018	10.2	0.328

a: All the enrolled studies applied AJCC/UICC 7th TNM staging system, except Wang YC's and Chouhei S’ studies using the 6th edition. OR: odds ratio; I^2^: inconsistency index; MSP: methylation-specific PCR; qMSP: quantitative methylation-specific PCR; H. pylori: *Helicobacter pylori*.

### Publication bias

**Figure 8:**
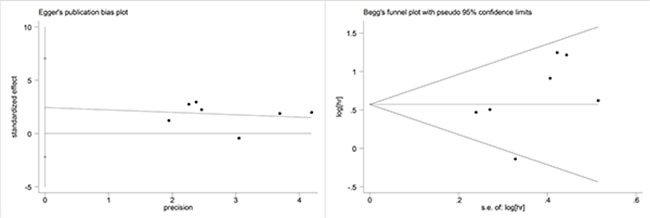
Funnel plot for the evaluation of potential publication bias in the impact of ctDNA on overall survival of GC patients (**A**) Begg's funnel plot; (**B**) Egger's funnel plot.

The publication bias was assessed in the association of ctDNA and OS in GC patients (Figure 8). Egger's tests and Begg's tests showed that publication bias was not significant for the enrolled studies (Egger's test: *p* = 0.233; Begg's test: *p* = 0.176).

## DISCUSSION

Traditional surgical/biopsy specimens are used in cancer diagnosis and considered as the gold standard for clinical examination. Pathology results according to surgical/biopsy specimens would also provide fundamental information for clinical decision-making. However, the limitations of specimen source directly from tumor, invasive procedure and delayed reflection for tumor dynamic change have restricted its appliance [25]. Therefore, liquid biopsy has been recently extensively investigated as one of the new diagnostic techniques [26].

Since the first report of fragmented DNA in the whole blood by Mandel and Metais [27] in 1948, cfDNA and ctDNA have been applied in a variety of disciplines. For example, detection of epidermal growth factor receptor (EGFR) T790M mutation in plasma is an effective method to determine EGFR status in NSCLC [5], providing a more expedient measure to predict resistance to EGFR tyrosine kinase inhibitors and prognosis [28]. However, the relationship between ctDNA and GC still remains unclear. Therefore, it is necessary to conduct comprehensive analysis illuminating the clinical utility of ctDNA in GC patient diagnosis and prognosis prediction.

In terms of test sensitivity, the present evidence showed no superiority of ctDNA over conventional protein biomarkers, such as CEA, CA125 and CA724, the combination of which had a diagnosis sensitivity of nearly 60–75% [29].Therefore, more accurate circulating gene targets need to be defined. On the other hand, the present ctDNA is considered to be more specific for certain types of cancer compared with normal tissue, mainly because the somatic cancer mutations have been identified by their presence in tumor DNA and absence in matched normal DNA [3]. Our analysis confirmed that detection of ctDNA had an obvious advantage in GC diagnosis specificity (specificity: 0.95, 95% CI 0.93–0.96). According to the suggested guidelines for the interpretation of the AUSROC value [30], ctDNA presence in patients had a relatively high diagnostic ability (AUC > 0.9) to indicate the risk of GC (Figure 5).

With regards to prognostic value, the detection of ctDNA in GC was significantly associated with both disease-free survival and overall survival. This finding will strongly strengthen the value of ctDNA in clinical management of patients with GC. The result of DFS part needs to be interpreted with caution due to limited enrolled study number. Moreover, detectable ctDNA was also associated with tumor size and TNM stage, which could be explained by the theory that circulating tumor genes are associated with tumor burden and invasion in patients [3].

The majority of the gene alteration types in this study were gene methylation/hypermethylation, which might result in inappropriate silencing of tumor suppressor genes. DNA methylation is relatively chemically stable and can be easily detected with a sensitivity of up to 1:1000 molecules [33]. In our meta-analysis, the methylation of *APC*, *P16* or *RASSF1A* genes were investigated in more than one independent studies. Taken *APC* as an example, it was first identified as the cause of the familial adenomatous polyposis syndrome and its dysfunction was closely associated with several gastrointestinal diseases [34]. Methylation-induced dysfunction of *APC* and subsequent activation of downstream pathways, such as the Wnt/β-catenin pathway, may be responsible for the aggressive tumor behavior [35].Consistent with the gene alteration types, the most common method of methylation detection is MSP or qMSP. The MSP technique is a useful procedure because of its high sensitivity and specificity [36]. Another gene alteration type described above is gene mutation, which could be detected by whole-genome sequence or genotyping technology [37].

Several limitations in this study should be addressed. First, the lack of a well-accepted ctDNA gene target in GC patients might contribute to the presence of bias. GC is considered as a malignancy with high histological and etiological heterogeneity. Therefore, more circulating genes customized by up-to-date molecular characterization would contribute to ctDNA detection and its clinical application in GC. Owing to the nature of our research, selection bias might occur with enrichment of studies reporting positive results. Furthermore, the difference in detection method and materials, such as PCR primers or the equipment applied, is also an important source of study bias. We also did not have enough information for comparing the ctDNA change before and after surgery, which could restrain the clinical application of ctDNA. Last, the majority of our enrolled studies came from East Asia countries, therefore, our conclusion might not be universal suitable.

Despite its preliminary nature, this study clearly indicated that ctDNA detection might be a specific, but low sensitive test in GC patients. The presence of ctDNA in GC patients predicted worse TNM stage and unfavorable survival. Before its wide application in GC patients, some concerns still need to be addressed, including more accurate molecule targets, suitable detection techniques. More prospective studies with consistent and standardized methodology are needed to further resolve these problems.

## MATERIALS AND METHODS

### Literature search

The search was conducted by searching the electronic databases Pubmed, Embase, Cochrane Library and Web of Science for all relevant papers published up to November 30, 2016. The following terms were used for searching: “Circulating tumor DNA” or “ctDNA” or “Blood/Serum/plasma DNA” and “gastric/stomach cancer” or “gastric/stomach tumor”. Article language was limited to English. Two researchers independently assessed the eligibility of the potential relevant studies by screening the titles and abstracts, and disagreements were solved by discussion. The references of all relevant papers were also checked to retrieve more eligible studies. The Preferred Reporting Items for Systematic Reviews and Meta-analysis (PRISMA) statement [38] was applied as the template for the searching process.

### Inclusion criteria

In this meta-analysis, eligible studies were selected according to the following inclusion criteria: (1) at least one of the diagnostic or prognostic value of ctDNA detection in GC patients was reported or able to be calculated from published data; (2) samples were collected from the peripheral blood; (3) the techniques and target gene were clearly stated in articles; and (4) studies must include negative controls.

### Exclusion criteria

The exclusion criteria were as follows: (1) studies published in languages other than English; (2) repeated or overlapping publications that included the same population and gene; (3) studies with a poor sample size (≤ 10); and (4) experiments only based on cell lines rather than clinical samples.

### Quality assessment

Two researchers independently reviewed and evaluated all eligible studies according to the Newcastle-Ottawa scale (NOS) [39]. This scale is an eight-item instrument that made assessment of patient population and selection, study comparability, and outcomes. And the ‘baseline characteristics of patients’ including patients’ gender, age and TNM stage were chosen as the ‘important factors’ in the Comparability section. We considered a study awarded five or more stars as a high-quality one.

The methodological quality of diagnosis part in this study was assessed by means of the revised Quality Assessment of Diagnostic Accuracy Studies (QUADAS-2) criteria [40]. The criteria consist of four key domains including patient selection, index test, reference standard and flow and timing. All four domains deal with the risk of bias while the first three domains also discuss concerns regarding applicability. Signaling questions are raised to assist measurement of bias, which are answered as “low risk”, “high risk” and “unclear risk”.

### Data extraction

The following items were extracted from the identified articles: title, name of the first author, publication year, region/country, clinicopathological features (i.e. gender proportion, sample size, TNM stage), detection details (i.e. target gene, genetic alteration type, detection method, time of sampling, sample volume), and accuracy of diagnostic trial (numbers of positive and negative patients for ctDNA detection). If the eligible studies provided survival data, then HR for OS or DFS and their 95% CIs were collected either directly from the articles or calculated using the methods illustrated by Parmar et al. [41] and Tierney et al [42].

### Statistical analysis

Diagnostic variables, such as sensitivity, specificity, likelihood ratios (i.e. positive likelihood ratios (PLR), negative likelihood ratios (NLR)), diagnostic ratios (DOR) and the summary receiver operating characteristic curve (SROC) were calculated and analyzed using the Meta-Disc software, version 1.4 [43]. The sensitivity was defined as the proportion of patients with ctDNA presence among all patients confirmed as GC. The specificity was defined as the proportion of patients with negative ctDNA detection among all negative control volunteers without GC. The PLR was calculated as sensitivity/ (1-specificity), while NLR was calculated as 1-sensitivity/specificity. Generally, a PLR > 5.0 and NLR < 0.2 was considered clinically significant. DOR was calculated as PLR/NLR, which indicated how much greater the chance of having GC is for the patients with ctDNA presence than for the ones without. The pooled HR and the 95% CIs for OS or DFS were analyzed by the Stata version 12.0 software (StatCorp, College Station, TX, USA). A significant heterogeneity was observed when *P* < 0.05 or *I*^2^ > 50%, and a random-effect model was used. Otherwise, a fixed-effect model was used.

National Nature Science Foundation of China (No.81272698, 81672319, 81602507) and Beijing Municipal Science and Technology Project (No.D131100005313010).

## SUPPLEMENTARY MATERIALS FIGURES AND TABLES


