# Supplementary material for: Diagnostic and prognostic value of circulating tumor DNA in gastric cancer: a meta-analysis
Source: Oncotarget. 2016 Dec 21;8(4):6330–40. doi: 10.18632/oncotarget.14064 (PMC5351635; doi:10.18632/oncotarget.14064)
Supplement: Supplementary file 1 [file oncotarget-08-6330-s001.pdf]

Diagnostic and prognostic value of circulating tumor DNA in gastric cancer: a meta-analysis

Supplementary Materials

Supplementary Table S1: Information of included genes and their main functions

	Full name	Gene function	Gene location
APC	APC	Signal transduction	5q22.2
BCL6B	B-cell CLL/lymphoma 6B	Signal transduction	17p13.1
E-cadherin	E-cadherin	Adhesion/invasiveness	16q22.1
MINT2	Amyloid beta precursor protein binding family A member 2	Signal transduction	15q11-q12
OSR2	Odd-skipped related transciption factor 2	Apoptosis/Proliferation	8q22.2
P16	Cyclin dependent kinase inhibitor 2A	Signal transduction	9p21
PCDH10	Protocadherin 10	Adhesion/Invasiveness	4q28.3
PPFIA3	PTPRF interacting protein alpha 3	Apoptosis/Proliferation	19q13.33
RASSF1A	Ras association domain family member 1	Apoptosis/Proliferation	3p21.3
RUNX3	Runt related transcription factor 3	Apoptosis/Proliferation	1p36
SFRP1	Secreted frizzled related protein 1	Signal transduction	8p11.21
SOX17	SRY-box 17	Adhesion/Invasiveness	8q11.23
SPG20	Spastic paraplegia 20	Signal transduction	13q13.3
TFPI2	Tissue factor pathway inhibitor-2	Signal transduction	7q22
TIMP3	TIMP metallopeptidase inhibitor 3	Adhesion/Invasiveness	22q12.3
VAV3	Vav guanine nucleotide exchange factor 3	Signal transduction	1p13.3
XAF1	XIAP associated factor 1	Apoptosis/Proliferation	17p13.1

Supplementary Table S2: Results of diagnosis quality assessment of included studies according to the QUADAS-2 tool criteria L: low risk of bias; H: high risk of bias; U: unclear risk of bias

Study	Risk of Bias				Applicability Concerns		
	Patients Selection	Index Test	Reference Standard	Flow and Time	Patients Selection	Index Test	Reference Standard
WK Leung[8]	L	L	U	L	H	L	L
Mohammad R.A.[9]	L	L	L	U	L	L	L
Wang YC[10]	L	L	U	L	L	L	L
Chouhei.S[11]	L	L	L	L	L	H	L
Kenji H.[12]	H	H	L	L	H	L	L
Ioanna B.[13]	H	L	L	L	H	L	L
Yang QF[14]	L	L	L	L	L	L	L
Zhi QL[15]	L	L	L	L	L	L	L
Han J[16]	L	L	L	L	L	L	L
Wu YC[17]	L	L	L	H	L	L	L
Yu JL[18]	L	L	L	L	H	L	L
Zhang H[19]	L	L	L	L	L	L	L
Chang L[20]	L	L	L	L	L	L	L
Ioanna B. (2015) [21]	H	L	L	H	H	L	L
Charinya P[22]	L	L	L	L	L	L	L
Li WH[23]	L	L	L	L	L	L	L

Supplementary Table S3: The assessment of quality of each study according to the newcastle-ottawa scale

Study	Selection(0-4)				Comparability		Outcome (0–3)			Total
	REC	Snec	AE	OINP	SCB	SCA	AO	FU	AFC	
WK Leung[8]	1	0	1	1	1	0	1	1	1	7
Mohammad R.A.[9]	1	1	1	1	1	1	0	0	0	6
Wang YC[10]	1	1	1	0	1	0	1	0	0	5
Chouhei.S[11]	1	1	1	0	1	0	1	0	0	5
Kenji H.[12]	1	1	1	0	1	1	0	0	0	5
Ioanna B.[13]	1	1	1	1	1	0	1	1	0	7
Yang QF[14]	1	1	1	0	1	0	1	1	0	6
Zhi QL[15]	1	1	1	1	1	1	1	1	0	8
Han J[16]	1	1	1	0	1	0	1	1	0	6
Wu YC[17]	1	1	1	0	1	1	1	1	0	7
Yu JL[18]	1	1	1	1	1	1	1	1	0	8
Zhang H[19]	1	1	1	0	1	0	1	1	0	7
Chang L[20]	1	1	1	0	1	0	1	0	0	5
Ioanna B. (2015)[21]	1	1	1	1	1	0	1	1	1	8
Charinya P[22]	1	1	1	1	1	1	1	1	0	8
Li WH[23]	1	1	1	0	1	0	1	0	0	5

REC: representativeness of the exposed cohort; SNEC: selection of the nonexposed cohort; AE: ascertainment of exposure; OINP: outcome of interest not presented in the start of study; SCB: study controls for basic characteristics; SCA: study controls for additional factor; AO: assessment of outcome; FU: follow-up; AFC: adequacy of follow up.
